# Assessment of craniofacial implants in restoring facial function and aesthetics

**DOI:** 10.6026/973206300211918

**Published:** 2025-07-31

**Authors:** Rohit Nandan, Akash Gopi, Shalabh Kumar, Samra Ashraf, Kshitiz Tyagi, Amisha Agarwal

**Affiliations:** 1 Department of Prosthodontics and Crown & Bridge, Teerthanker Mahaveer Dental College, Moradabad, Uttar Pradesh, India

**Keywords:** Aesthetics, craniofacial implants, facial function, patient satisfaction, rehabilitation

## Abstract

The effectiveness of craniofacial implants in restoring facial function and aesthetics in patients with congenital anomalies, trauma, tumors, or other craniofacial deformities is of interest. Fifty patients aged 18 and above who had received craniofacial implants were assessed over 12 months at three intervals: pre-surgery, 6 months post-surgery and 12 months post-surgery. Clinical evaluations, standardized functional and aesthetic scales and patient-reported outcomes were used to measure implant success. Results showed significant improvements in facial and oral functions, aesthetic outcomes, patient satisfaction and pain reduction. Although minor complications such as implant failure and infection were observed, the overall success rate was high. Thus, the role of craniofacial implants in improving patients' quality of life is shown, while emphasizing the need for long-term research to refine rehabilitation techniques.

## Background:

Craniofacial malformations are congenital anomalies that arise from various causes, including chromosomal, environmental, Mendelian, multifactorial, and pathogenetic mechanisms such as malformations, deformations, disruptions, or dysplasias [1]. These defects may present as isolated issues or as part of a syndrome or sequence [2]. Acquired craniofacial defects can result from degenerative processes, infections, trauma, burns, or cancer treatments, often leading to psychological and social challenges [3]. Treatment options include plastic surgery and prosthetic rehabilitation, with autologous reconstruction often requiring multiple stages to achieve satisfactory cosmetic outcomes [4]. Patient comorbidities, such as scarring and vascular damage from radiotherapy, complicate reconstructive efforts [5]. Prosthetic reconstruction is a viable alternative, especially when autologous methods fail. Prostheses can also assist with cancer monitoring by allowing access to the orbit and reducing recurrence risks [6]. Skin adhesives, spectacles, and anatomical undercuts are commonly used for prosthetic retention, though adhesives can cause skin reactions, edge deformation, and loss of adhesion, leading to suboptimal outcomes [7]. Intraosseous osseointegration implants have improved functional and aesthetic outcomes in patients with extensive craniofacial defects, enhancing their quality of life [8].

Implant therapy is removable and adaptable, but patient-specific considerations must be evaluated [9]. Over the past decade, additive manufacturing (AM), also known as 3D printing, has demonstrated promise in creating personalized implants, utilizing materials such as metals, ceramics, and biocompatible polymers like PEEK, PEKK, and PMMA for customized craniofacial implants [10]. Despite the current limitations, advancements in digital technologies and intraoperative image-guided navigation offer promising prospects for improving craniofacial rehabilitation [11]. Therefore, it is of interest to explore the potential of 3D printing in enhancing craniofacial reconstruction.

## Methodology:

This prospective, observational study aims to assess the effectiveness of craniofacial implants in restoring facial function and aesthetics in patients who have undergone craniofacial implant procedures due to congenital malformations, trauma, tumors, or other craniofacial deformities. The study will include patients aged 18 years and above who have received craniofacial implants within the last 6 months and have provided informed consent. Patients with severe systemic diseases or conditions that prevent follow-up for the study duration will be excluded. A target sample size of 50 patients will be selected to ensure sufficient statistical power to detect clinically significant outcomes. Data will be collected at three time points: pre-surgery, 6 months post-surgery and 12 months post-surgery. Clinical evaluations will include a detailed examination of the implant site to assess integration with surrounding tissue, facial and oral function using standardized tests like the Facial Function Scale (FFS) and the Oral Function Scale (OFS) and aesthetic outcomes using the Visual Analog Scale (VAS). Patient-reported outcomes will be measured through questionnaires such as the Craniomaxillofacial Disability Index (CMDI) and the Patient-Reported Outcomes Measurement Information System (PROMIS) to assess quality of life, facial appearance and functionality. Pain levels will be assessed using the Visual Analog Scale for Pain (VAS-Pain). Additionally, preoperative and postoperative CT scans or 3D imaging will be used to evaluate implant placement and integration, while radiographs will be analyzed for osseointegration and implant stability. The primary outcomes will focus on improvements in facial function, aesthetic outcomes and patient satisfaction. Secondary outcomes will include complication rates such as implant failure, infection and the need for additional interventions. Statistical analysis will be conducted using SPSS software, with descriptive statistics to summarize patient demographics and clinical outcomes. Paired t-tests or Wilcoxon signed-rank tests will be used to compare preoperative and postoperative results and chi-square tests will be applied to evaluate categorical variables. Ethical approval will be sought from the institutional review board (IRB) and written informed consent will be obtained from all participants to ensure voluntary participation and confidentiality. Although the study has limitations, including a short follow-up period and a sample size that may not fully represent all patient demographics, it aims to provide important insights into the role of craniofacial implants in improving facial function and aesthetics. This research could help inform future practices and improve craniofacial rehabilitation techniques.

## Results:

This prospective study included 50 patients who underwent craniofacial implant procedures to assess the restoration of facial function and aesthetics. The clinical outcomes were evaluated at three time points: pre-surgery, 6 months post-surgery and 12 months post-surgery. The patients included had various craniofacial deformities caused by congenital malformations, trauma, tumor treatments, or other factors. The study measured facial function, oral function, aesthetic outcomes, patient satisfaction and pain levels, with significant improvements observed across all parameters. Pre-surgery, the average facial function score was 3.2, indicating moderate facial mobility. Post-surgery, the score improved to 4.5 at 6 months and further to 5.2 at 12 months. Oral function showed similar progress, with the pre-surgery score at 2.8, increasing to 4.2 at 6 months and 4.7 at 12 months. Aesthetic outcomes, initially scored at 2.1 pre-surgery, improved to 4.3 at 6 months and 4.7 at 12 months. Patient satisfaction, measured by the PROMIS scale, rose from 4.3 pre-surgery to 5.5 at 6 months and 5.8 at 12 months. Pain levels, initially high at 6.5, decreased to 3.1 at 6 months and further to 2.7 at 12 months (Table 1 - see PDF). The study also identified complications, with 1 patient experiencing implant failure, 2 patients experiencing infection and 3 patients facing issues related to prosthetic retention and adjustments. Despite these complications, the overall success of the craniofacial implants was high, demonstrating their efficacy in restoring facial function and aesthetics. These findings suggest that craniofacial implants are an effective solution for patients with facial deformities, improving both functional and aesthetic outcomes. The study highlights the importance of craniofacial implants in enhancing the quality of life for patients, although further research with larger sample sizes and longer follow-up periods is needed to assess the long-term outcomes and potential improvements in treatment fully. Details of complications observed during the study have been shown in Table 2 (see PDF).

## Discussion:

This prospective study assessed the effectiveness of craniofacial implants in restoring both facial function and aesthetics in patients with craniofacial deformities. The results showed significant improvements across various clinical and patient-reported outcomes. Facial function, oral function, aesthetic outcomes and patient satisfaction all improved post-surgery, with reductions in pain levels. These findings suggest that craniofacial implants can significantly enhance both functional capabilities and the aesthetic appearance of patients suffering from facial deformities. In our study, patients exhibited improvements in facial and oral function, aesthetic outcomes, patient satisfaction and a reduction in pain levels post-surgery. These results are consistent with the findings of Subramaniam *et al.* [12] who observed high success rates in implants placed for congenital deformities and temporal regions. Notably, orbital implants in their study had a lower survival rate of 63.3%, highlighting the complexity and challenges associated with reconstructing the orbital region. Both studies underscore the importance of careful patient selection and individualized treatment planning in achieving optimal outcomes with craniofacial implants. While our study provides valuable information on short-term improvements in function and aesthetics, the long-term data from Subramaniam *et al.* offer a more comprehensive understanding of the factors influencing implant success and failure over extended periods. In our prospective study, we observed significant improvements in oral function, facial aesthetics and patient satisfaction following craniofacial implant procedures. Specifically, oral function scores increased from 2.8 pre-surgery to 4.7 at 12 months post-surgery. These findings align with those of Schmidt *et al.* [13], who reported that patients with craniofacial disorders (CD) exhibited reduced masticatory efficiency compared to healthy controls, with a higher number of larger food particles indicating less efficient chewing. Their study utilized a standardized food model test to assess masticatory efficiency in orthodontic patients aged 7-21 years. Patient satisfaction in this study was also significantly improved, our patients reported a significant increase in satisfaction, with PROMIS scores improving from 4.3 pre-surgery to 5.8 at 12 months. These results reflect the high degree of satisfaction patients experience when their facial function and aesthetics are restored through craniofacial implants. Pain reduction is a crucial aspect of the rehabilitation process and our study showed a substantial decrease in pain levels, from 6.5 pre-surgery to 2.7 at 12 months. The improvement in pain levels in our study further supports the notion that craniofacial implants contribute to enhanced post-operative comfort and quality of life. Despite the overall success of craniofacial implants in this study, complications did arise, including implant failure, infection and prosthetic retention issues. The occurrence of implant failure in 1 patient and infection in 2 patients is consistent with findings from Alberga *et al.* [14], who also reported complications such as infections and implant failures in their study on craniofacial implants. The complications observed in our study were manageable, with most patients recovering with minor interventions. However, these complications highlight the need for careful post-surgical monitoring and intervention to ensure the long-term success of the implants. Furthermore, the complication rates in our study were similar to those observed in previous research, which also reported minor issues with implant failure and infections. However, despite these challenges, the overall success of craniofacial implants in restoring function and aesthetics remained high, which aligns with Dutta *et al.* [15], who emphasized the importance of careful patient selection and follow-up care in minimizing complications. Subramaniam *et al.* [16] conducted a long-term study on craniofacial implants, focusing on their effectiveness in restoring facial defects. The study found that craniofacial implants demonstrated high success rates for patients with congenital deformities, particularly in the temporal and facial regions. However, the authors also identified challenges related to orbital implants, which exhibited a lower survival rate of 63.3%. The results underline the importance of personalized treatment plans and careful patient selection to achieve optimal outcomes. This aligns with the current study, which also highlights the necessity of patient-specific strategies for successful rehabilitation through craniofacial implants. Goiato *et al.* [17] examined the success of craniofacial implants in facial rehabilitation. Their study reported substantial improvements in both functional and aesthetic outcomes for patients undergoing craniofacial implant procedures. Patient satisfaction was notably higher post-surgery, reflecting the positive psychological and emotional effects of enhanced facial appearance and function. The findings of Goiato *et al.* are consistent with our study, where patient satisfaction and improvement in facial aesthetics were significantly enhanced following craniofacial implant procedures. This reinforces the idea that craniofacial implants not only restore function but also have a profound impact on the patients' psychological well-being. Kauke-Navarro *et al.* [18] provided a comprehensive review of facial implant materials used in craniofacial surgery. They discussed the balance between the aesthetic goals of craniofacial implants and the scientific considerations regarding material selection. The study emphasized the importance of choosing materials that optimize both cosmetic outcomes and long-term durability, particularly when reconstructing complex facial regions. This review complements our study by underscoring the critical role of material choice in the success of craniofacial implants. In line with Kauke-Navarro *et al.* [18] our findings suggest that while the clinical outcomes for craniofacial implants are promising, the material selection remains a pivotal factor in the overall success of these procedures.

## Conclusion:

The efficacy of craniofacial implants in restoring both facial function and aesthetics is shown. The improvements in all measured outcomes, along with the relatively low complication rate, suggest that craniofacial implants are a valuable treatment option for patients with craniofacial deformities. However, there is a necessary to refine the techniques, reduce complications and further enhance patient satisfaction.

## Financial support:

Nil
